# Mitochondrial haplotype diversity and population dynamics of the sugarcane borer, *Diatraea saccharalis* (Lepidoptera: Crambidae), in Jamaica

**DOI:** 10.1093/jee/toag111

**Published:** 2026-04-27

**Authors:** Damion O Neath, Paula F Tennant, Andrea L Joyce, Dwight E Robinson

**Affiliations:** Department of Life Sciences, University of West Indies, Mona, Jamaica; Research Projects Branch, Sugar Industry Authority, Mandeville, Jamaica; Department of Life Sciences, University of West Indies, Mona, Jamaica; Caribbean Centre for Research in Bioscience, University of West Indies, Mona, Jamaica; Sierra Nevada Research Institute, University of California Merced, Merced, CA, USA; Department of Life Sciences, University of West Indies, Mona, Jamaica

**Keywords:** *Diatraea saccharalis*, COI, genetic diversity, population structure, Jamaica

## Abstract

The sugarcane borer, *Diatraea saccharalis* (Fabricius) (Lepidoptera: Crambidae), is a major pest of sugarcane in the Caribbean, yet little is known about the genetic structure of populations in the region. In this study, the genetic diversity and phylogeographic relationships of *D. saccharalis* in Jamaica, a major island in the Caribbean, were evaluated based on mitochondrial cytochrome oxidase subunit I (COI) sequences. A total of 25 haplotypes were identified among 239 individuals sampled from 25 locations across the island. Overall genetic diversity was moderate, with site-level diversity ranging from very low to high. The widespread occurrence of a single dominant haplotype, along with a star-like haplotype network and low significant population differentiation, suggested high gene flow across the island. Analysis of molecular variance (AMOVA) showed that most genetic variation (94.47%) occurred within populations, with only 5.53% between populations. Neutrality tests produced significantly negative values, consistent with recent demographic expansion. Phylogenetic analysis placed the Jamaican haplotypes in a distinct Caribbean Clade closely associated with populations from Florida, indicating regional genetic structuring. These findings provide important insights into the evolutionary history of *D. saccharalis* and highlight the value of incorporating population genetic data into sustainable pest management strategies for the Caribbean.

## Introduction

The sugarcane borer, *Diatraea saccharalis*, is a principal pest of sugarcane in the Americas, causing significant losses both in the field and at the factory level (Metcalfe 1969, Ferreira et al. 2018). Damage in the field is observed at 2 stages: in young plants, where the borer feeds on the central meristem, resulting in a condition known as “dead heart” (Capinera 1969); and in mature plants, where the pest feeds internally, creating galleries in the sugarcane stalks and allowing fungi such as *Fusarium verticilliodes* (the causal agent of red rot) to infect the plant. At the factory level, losses are reflected in reduced sucrose content and lower juice purity. An inverse relationship exists between the percentage of bored internodes caused by *D. saccharalis* and key metrics such as sugar per acre, juice purity, stalk weight, cane per acre, sucrose per ton of sugarcane, and overall sucrose content (Legaspi et al. 1999).

The sugarcane borer is native to and is restricted to the New World, with a distribution ranging from the southern United States through Central America and the Caribbean, and throughout much of South America (Bleszynski 1969, Pemberton and Williams 1969, Edde 2022). Female moths lay eggs on the underside of cane leaves near the mid-rim. The eggs hatch within 4 to 9 days, and the larvae, which last for 25 to 30 days, emerge (Holloway et al. 1928). The larvae then pupate within the cane stalk for 6 to 9 days before the adults emerge, living for 3 to 8 days (Holloway et al. 1928, Capinera 1969). Typically, species identification relies on the inspection of adult genitalia (Box 1931, Solis and Metz 2016). However, identification based on general morphology is difficult because *D. saccharalis* exhibits considerable morphological variation in size and coloration across its geographic range (Silva-Brandão et al. 2015a).

Several studies in recent years have used PCR-based techniques including DNA barcoding to identify sugarcane borer species and to assess the genetic diversity and population structure across the Americas (Joyce et al. 2014, 2016, Silva-Brandão et al. 2015a, Pavinato et al. 2018, Francischini et al. 2019, Fogliata et al. 2022). Genetic diversity studies of *Diatraea saccharalis* populations in Brazil revealed moderate population differentiation and sex-linked genetic variation (Heideman et al. 2010, Lopes et al. 2014). A study in Brazil sequenced a portion of the mtDNA COI gene of sugarcane borers collected from both corn and sugarcane and found a strong genetic structure and significant correlation between pairwise genetic distances and geographic distance (Silva-Brandão et al. 2015a). Another, study in Brazil analyzed a different region of the mtDNA COI gene and reported relatively little spatial divergence in the *D. saccharalis* population, although the results were consistent with previous studies that identified distinct clusters in South American and Southern U.S. populations (Francischini et al. 2017). This distinct population structuring of the sugarcane borer was further supported by genome-wide single nucleotide polymorphism (SNP) markers, which provided robust evidence of historical gene flow and inferred migration pathways of the species across the region over several centuries (Francischini et al. 2019). Conversely, in Argentina, *D. saccharalis* exhibited no clear genetic structuring by geographic location or host plant when analyzed using SNPs markers, although high genetic variation was observed between populations in Argentina and Brazil when COI markers were compared (Fogliata et al. 2022).

Microsatellite analyses of *D. saccharalis* populations in Brazil revealed 2 significant genetic clusters; one corresponding to pronounced spatial differentiation and the other potentially associated with cryptic host plant differentiation (Pavinato et al. 2018). Geographic differentiation was also observed when COI gene fragments from samples in El Salvador were compared with those from South and North America, supported by both mtDNA COI and amplified fragment length polymorphism (AFLP) analysis (Joyce et al. 2016). In the Southern U.S., analysis of a segment of the COI gene, revealed three genetically distinct clusters of *D. saccharalis* across the Western Hemisphere, suggesting a cryptic species complex in Florida (Joyce et al. 2014). Further analysis suggested that comparisons between Florida and Caribbean populations may clarify the origin of Florida’s sugarcane stalk borer populations.

Despite the wide geographic distribution, extensive host plant range, and apparent genetic geographic differentiation, *D. saccharalis* is considered a single species. Management practices are similar across its range, with biological control being the preferred and most successful method of managing sugarcane borer in the Caribbean (Baker et al. 1992) and host plant resistance being an important management technique as well. However, information on *D. saccharalis* populations in the Caribbean remains limited. In particular, the population in Jamaica has not been investigated, and the genetic composition and potential variability of local populations remain unknown. Based on morphological characteristics*, D. saccharalis* is the only species of the genus recorded in Jamaica. In 2023, infestation levels were reported at 9.25%, 4.46%, and 2.68% in the sugarcane growing parishes of St. Ann, Clarendon, and St. Catherine, respectively. This has raised concerns among cane growers, since cane payment is contingent on the purity of the extracted juice. It is estimated that in Jamaica, for every 1% of internode damage caused by the borer, there is a 0.23% reduction in juice quality (Chinloy 1955). Given the economic impact of borer infestation, accurate identification and an understanding of the pest’s population dynamics are critical for effective management and for selecting appropriate Integrated Pest Management (IPM) strategies.

This study aims to assess the genetic diversity and structure of *D. saccharalis* populations across Jamaica’s cane-growing areas using mitochondrial COI gene sequences. The use of molecular markers, such as COI, is well suited for studying the Jamaican populations, as these markers have been widely applied in recent studies alongside newer approaches, and will allow comparisons with previous studies across the Americas.

## Materials and Methods

### Sugarcane Borer Collection

**Fig. 1.**
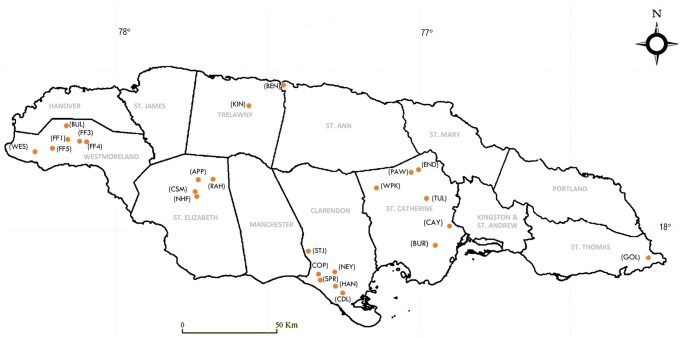
Map of Jamaica showing the sampling locations for *Diatraea saccharalis* collections across major sugarcane growing regions. Each point represents a site included in the study. Location code: Caymanas Estate (CAY), Frome Farms 1, 3, 4, and 5 (FF1, FF3, FF4, FF5), Appleton (APP), Hannif Farm (HAN), New Yarmouth (NEY), Bull Head (BUL), Tulloch Estate (TUL), Clarendon Distillers (CDL), Bursingh Farm (BUR), Casa Marantha (CSM), Springfield (SPR), Worthy Park (WPK), Raheen (RAH), Palm/Wallen (PAW), Kinloss (KIN), Endfield (END), St Jago (STJ), Bengal Farm (BEN), Golden Grove (GOL), Westmoreland (WES), Copper Farm (COP), and New Hope Farm (NHF).

Sugarcane borer larvae were collected from 7 sugarcane-growing parishes across 25 localities in Jamaica between 2019 and 2022 (Fig. 1). Fields were inspected for signs of *D. saccharalis* infestation, including exit and entry holes in cane stalks, frass associated with borer feeding, and in some instances, the presence of “dead heart” symptoms. Damaged stalks were excised and split longitudinally to retrieve borer larvae or pupae which subsequently placed in plastic containers for transportation to the laboratory. To reduce the risk of oversampling sibling larvae, samples were collected at least 1 m apart when surveying the same field. The GPS coordinates of each farm were recorded.

Individual larvae were reared to adulthood at the Sugar Industry Authority Entomology Laboratory on an artificial diet (USDA formula) provided by Frontier Agricultural Services, USA. Parasitized larvae were recorded and discarded, while pupated larvae were allowed to develop into adults. Upon emergence, adults were preserved in 100% ethanol and stored at −4 °C until DNA extraction. Specimens collected from the same locality were stored together and assigned a unique code based on their collection site.

### Insect DNA Extraction, Mitochondrial COI Amplification, and Sequencing

Total genomic DNA was extracted from the thoracic region of adult moths, whole pupae or the larvae (with internal organs removed) using a modified cetyltrimethylammonium bromide (CTAB) method (Doyle and Doyle 1987). DNA purity and concentration were evaluated by electrophoresis on 1% agarose gels and UV/Vis spectroscopy (NanoDrop, Thermo Fisher Scientific, USA).

A partial fragment of the mitochondrial cytochrome oxidase subunit I (COI) gene was amplified by polymerase chain reaction (PCR) using the primers LepF (5′-ATTCAACCAATCATAAAGATATTGG-3′) and LepR (5′-TAAACTTCTGGATGTCCAAAAAATCA-3′) (Hajibabaei et al. 2006). Each 25 µl PCR reaction contained 12.5 µl (1×) of GoTaq Green Master Mix (Promega, USA), 0.4 µl (0.12 µM) of each primer (Integrated DNA Technologies, USA), 2 µl (5.6 ng/µl) of template DNA, and 9.7 µl of sterile distilled water. PCR amplifications were performed in an Applied Biosystems 2720 Thermal Cycler under conditions described by EPPO (2021): initial denaturation at 95 °C for 15 min, followed by 5 cycles of 45 s at 95 °C, 45 s at 45 °C, and 45 s at 72 °C, then 35 cycles of 45 s at 95 °C, 45 s at 49 °C, and 45 s at 72 °C, with a final extension of 7 min at 72 °C. Successful amplification was confirmed by 1% agarose gel electrophoresis. PCR products were then purified and sequenced bidirectionally using the forward and reverse primers on an Applied Biosystems ABI 3730XL DNA sequencer at the University of California, Merced.

### Genetic Diversity, Phylogenetic Analysis, and Population Structure

Consensus sequences generated from sense and antisense chromatograms using the CAP3 tool in Unipro UGENE (Okonechnikov et al. 2012) were edited and assembled in BioEdit (Hall 1999). Sequence alignment and nucleotide composition analysis were performed using MEGA7 (Kumar et al. 2016). Pairwise genetic distances between *Diatraea saccharalis* populations were calculated using the Kimura 2-parameter model in MEGA7. Additionally, a maximum likelihood (ML) phylogenetic tree was constructed in MEGA7 using COI haplotypes from Jamaica alongside 40 *D. saccharalis* sequences from the North and South American regions [USA (FL, LA, TX), Mexico, El Salvador, Bolivia, Brazil, Argentina] obtained from GenBank and BOLD databases (see Supplementary Table S1). The tree was constructed under the Tamura 3-parameter model with a gamma distribution (T92 + G) and node support was assessed with 1,000 bootstrap replicates. Pairwise distances between the Jamaican samples and other regional samples were also generated using the Kimura 2-parameter in MEGA7. All sequences were aligned to a uniform length, and no missing data or gaps were present in the final dataset.

For all phylogenetic trees, outgroup sequences from other *Diatraea* species were retrieved from GenBank. These included *D. considerata* (JQ888365.1), *D. grandiosella* (JQ888348.1), *D. lisetta* (KJ657593.1), *D. mitteri* (KR070998.1), and *D. crambidoides* (KR070995.1).

Nucleotide diversity (*π*), haplotype diversity (*h*), and the number of haplotypes were estimated using DnaSP v5 (Librado and Rozas 2009). Genealogical relationships among mitochondrial haplotypes were constructed using PopArt (Leigh and Bryant 2015) inferred with the network of median-joining method. Neutrality tests, including Tajima’s *D* (Tajima 1989) and Fu’s *Fs* (Fu 1997), were also performed in DnaSP v5. Genetic diversity among and within populations was assessed by analysis of molecular variance (AMOVA) using Arlequin v3.5 (Excoffier and Lischer 2010). Population genetic divergence (*F*_ST_) and pairwise *F*_ST_ values were derived from the AMOVA results. Pairwise genetic distances and *F*_ST_ values were visualized using heatmaps in Microsoft Excel to further to facilitate the interpretation of genetic differentiation patterns among populations. To estimate gene flow among populations, the number of migrants per generation (*N_m_*) was inferred from Wright’s Island model (Wright 1951) using the formula:


Nm = (1 - FST)/(4 × FST)

where *F*_ST_ represents the overall genetic differentiation among populations. The *F*_ST_ value used in this calculation was obtained from the analysis of molecular variance (AMOVA).

To test for isolation by distance, a Mantel test was conducted in R statistics. The pairwise genetic distance matrix values were compared with the corresponding geographic distances (in kilometers) between sampling sites. The Mantel test was performed with 9,999 permutations to assess the statistical significance of the correlation. The Mantel correlation coefficient (*r*) and associated *P* value were used to evaluate the relationship between genetic and geographic distances.

## Results

### Sample Collection and Sequence Analysis

A total of 481 *D. saccharalis* (F.) larvae were collected from 25 localities across 7 sugarcane-producing parishes in Jamaica between 2019 and 2022 (Fig. 1). A 623 base pair (bp) fragment of the mitochondrial cytochrome oxidase subunit I (COI) gene was successfully sequenced from a subset of 239 adult specimens. All sequences obtained in this study were identified as *Diatraea saccharalis*. They matched sequences of *D. saccharalis* from across the Americas with high similarity (≥99%) in nucleotide NCBI BLAST.

Of the 623 bp sequences analyzed, 599 sites were conserved, and 31 sites (4.9%) were variable. Among the variable sites, 22 were singleton polymorphic sites, and 9 were parsimony-informative. The average nucleotide composition across all sequences was 31.3% adenine (A), 37.2% thymine (T), 15.4% guanine (G), and 16.1% cytosine (C), demonstrating a pronounced A/T bias. This compositional bias is consistent with patterns commonly observed in Lepidoptera and aligns with previously reported mitochondrial genome characteristics of *D. saccharalis* (Taylor et al. 1993, Li et al. 2011). All COI sequences generated in this study have been deposited in the GenBank database under accession numbers PZ245593–PZ245831.

### Genetic Diversity and Haplotype Distribution

A total of 25 mitochondrial COI haplotypes were identified across the sampled Jamaican *D. saccharalis* populations (Table 1), designated Hap_1 through Hap_25. Hap_1 was the most prevalent, occurring in 185 individuals and present at all sampling locations. In contrast, 22 haplotypes were unique to individual localities. The New Yarmouth (NEY) site exhibited the highest haplotype richness, with 7 haplotypes in total, including 4 unique haplotypes. Hap_2 was the second most common, observed in 23 individuals and detected at 13 localities. Only a single haplotype (Hap_1) was recorded at Frome Farm 1 (FF1), Frome Farm 5 (FF5), Tulloch Estate (TUL), Worthy Park (WPK), Bengal Farm (BEN), and Westmoreland (WES).

**Table 1.** Sample size (*N*), haplotype, number of haplotypes (*n*), haplotype diversity (*h*), nucleotide diversity (*π)*, and neutrality test results for *Diatraea saccharalis* in various locations across Jamaica

Locality	*N*	Haplotype	*n*	*h* ± SD	π	Tajima’s *D*
**Caymanas Estate (CAY)**	15	Hap_1 (10), Hap_2 (2), **Hap_6** (2), **Hap_7** (1)	4	0.552 ± 0.137	0.0010	−0.948
**Frome, Farm 1 (FF1)**	13	Hap_1 (13)	1	0.000	0.0000	0.000
**Frome, Farm 3 (FF3)**	12	Hap_1 (9), **Hap_10** (2), **Hap_11** (1)	3	0.439 ± 0.158	0.0008	−0.849
**Frome, Farm 4 (FF4)**	2	Hap_1 (1), **Hap_12** (1)	2	1.000 ± 0.500	0.0016	0.000
**Frome, Farm 5 (FF5)**	1	Hap_1 (1)	1	0.000	0.0000	0.000
**Appleton (APP)**	12	Hap_1 (7), Hap_2 (4), **Hap_25** (1)	3	0.591 ± 0.108	0.0011	−0.047
**Hannif Farm (HAN)**	12	Hap_1 (10), Hap_2 (1), Hap_15 (1)	3	0.318 ± 0.164	0.0005	−1.451
**New Yarmouth (NEY)**	11	Hap_1 (4), Hap_2 (2), Hap_15 (1), **Hap_17** (1), **Hap_18** (1), **Hap_19** (1), **Hap_20** (1)	7	0.873 ± 0.089	0.0022	−1.358
**Bull Head (BUL)**	12	Hap_1 (9), Hap_2 (2), **Hap_3** (1)	3	0.439 ± 0.158	0.0009	−0.381
**Tulloch Estate (TUL)**	12	Hap_1 (12)	1	0.000	0.0000	0.000
**Clarendon Distillers (CDL)**	11	Hap_1 (9), Hap_2 (2)	2	0.327 ± 0.153	0.0005	−0.100
**Bursingh Farm (BUR)**	12	Hap_1 (7), **Hap_4** (5)	2	0.530 ± 0.076	0.0017	1.757
**Casa Marantha (CSM)**	7	Hap_1 (5), Hap_2 (1), **Hap_5** (1)	3	0.524 ± 0.209	0.0009	−1.237
**Springfield (SPR)**	12	Hap_1 (8), Hap_2 (3), **Hap_23** (1)	3	0.530 ± 0.136	0.0012	−0.828
**Worthy Park (WPK)**	12	Hap_1 (12)	1	0.000	0.0000	0.000
**Raheen (RAH)**	12	Hap_1 (9), Hap_2 (2), **Hap_22** (1)	3	0.439 ± 0.158	0.0008	−0.849
**Palm/Wallen (PAW)**	7	Hap_1 (6), **Hap_21** (1)	2	0.286 ± 0.196	0.0046	−1.609^a^
**Kinloss (KIN)**	4	Hap_1 (3), Hap_2 (1)	2	0.500 ± 0.265	0.0008	−0.612
**Endfield (END)**	11	Hap_1 (9), Hap_2 (1), **Hap_9** (1)	3	0.345 ± 0.172	0.0006	−1.429
**St Jago (STJ)**	10	Hap_1 (8), Hap_2 (1), **Hap_24** (1)	3	0.378 ± 0.181	0.0012	−1.034
**Bengal Farm (BEN)**	12	Hap_1 (12)	1	0.000	0.0000	0.000
**Golden Grove (GOL)**	11	Hap_1 (8), **Hap_13** (2), **Hap_14** (1)	3	0.473 ± 0.162	0.0011	−1.113
**Westmoreland (WES)**	5	Hap_1 (5)	1	0.000	0.0000	0.000
**Copper Farm (COP)**	5	Hap_1(3), Hap_2 (1), **Hap_8** (1)	3	0.700 ± 0.218	0.0013	−0.972
**New Hope Farm (NHF)**	6	Hap_1 (5), **Hap_16** (1)	2	0.333 ± 0.215	0.0005	−0.933
**Total**	239		25	0.392 ± 0.040	0.0009	−2.476^a^

Unique haplotypes resented in bold and number of each haplotype in parenthesis; *N*, sample number; *n*, number of haplotypes; *h*, haplotype diversity; *π*, nucleotide diversity; SD, standard deviation.

a *P* ≤ 0.0500.

Haplotype diversity (*h*) and nucleotide diversity (*π*) were calculated for all 25 localities. Haplotype diversity ranged from 0.000 to 1.000 ± 0.500 (Table 1). The highest haplotype diversities were observed at Frome Farm 4 (FF4) (*h *= 1.000 ± 0.500) and New Yarmouth (NEY) (*h *= 0.873 ± 0.089). Nucleotide diversity (*π*) ranged from 0.0000 to 0.0046, with the highest value recorded at Palm/Wallen (PAW) (*π*  =  0.0046). Both haplotype and nucleotide diversity were zero at FF1, FF5, TUL, WPK, BEN, and WES, consistent with the presence of a single haplotype at these locations. Overall genetic diversity for the entire population was moderate, with haplotype diversity (*h*) = 0.392 ± 0.040 and nucleotide diversity (*π*) = 0.0009.

### Phylogenetic Analysis and Haplotype Network

**Fig 2.**
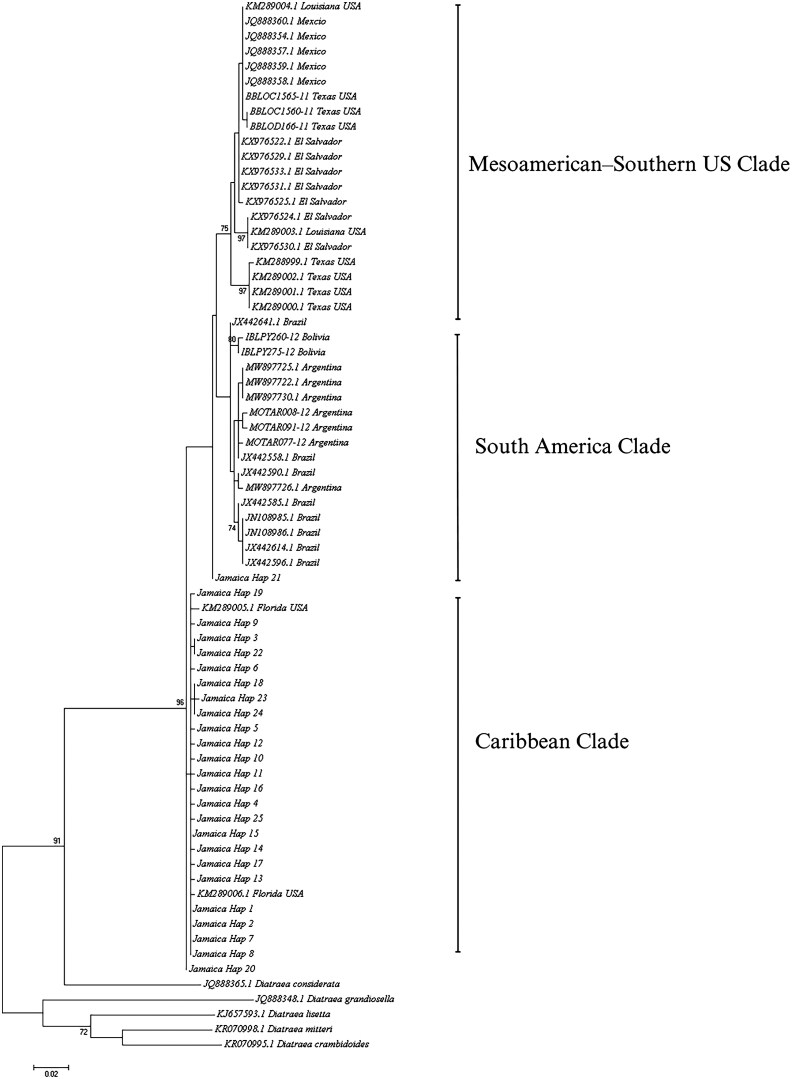
Phylogenetic tree based on maximum-likelihood (ML) inferred with Kimura 2-parameter using 1,000 bootstrap replicates of Jamaican COI haplotypes and 70 *Diatraea saccharalis* COI sequences from the North and South American regions [USA (Florida, Louisiana, Texas), Mexico, El Salvador, Bolivia, Brazil, Argentina]; *Diatraea considerata*, *Diatraea grandiosella*, *Diatraea lisetta*, *Diatraea mitteri*, and *Diatraea crambidoides* obtained from GenBank used as the outgroup.

The maximum likelihood (ML) phylogenetic tree constructed using the COI gene and the Tamura 3-parameter model revealed a well-supported monophyletic clade comprising the majority of Jamaican haplotypes (Hap_1–Hap_19 and Hap_25), with a bootstrap support value of 99 (Fig. 2). Despite the overall clustering, several branches within the tree remained unresolved, indicating low divergence among haplotypes. Hap_20 formed a distinct clade separate from the main group, although this branching was only moderately supported (bootstrap value >75). Hap_21 was more divergent, branching off earlier in the tree and forming a sister lineage to the main clade.

**Fig 3.**
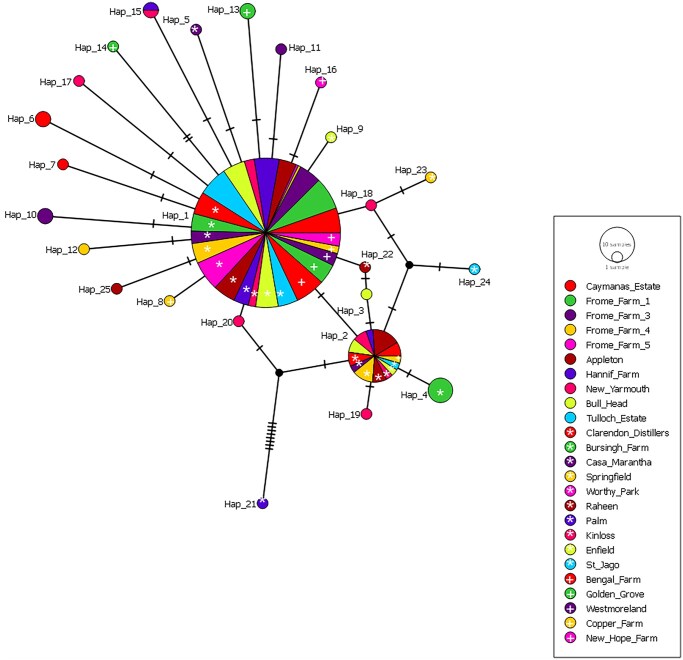
Haplotype network of *Diatraea saccharalis* based on COI gene sequences from 25 identified haplotypes across sampled locations in Jamaica, constructed using the neighbor-joining method in PopArt. Each haplotype is represented by a circle, with the colors indicating specific locations. The size of each circle reflects the frequency of the haplotype, while the colors show where the haplotype was found. Hatch marks along the connecting lines represent mutational steps between haplotypes.

The haplotype network generated using the neighbor-joining method was largely congruent with the ML tree and displayed a star-like topology centered around Hap_1 (Fig. 3). Eighteen haplotypes were connected directly to Hap_1 by 1 to 2 mutational steps, underscoring its central role in the network. Hap_21, identified at Palm/Wallen (PAW), was the most divergent, separated from Hap_1 by 10 mutational steps.

Neither the phylogenetic tree nor the haplotype network revealed a clear geographic structure or spatial partitioning among haplotypes, suggesting limited population differentiation across sampling locations.

### Pairwise Genetic Distance and Genetic Differentiation

Pairwise genetic distances and genetic differentiation (*F*_ST_) among *D. saccharalis* populations were visualized in a heatmap (see Supplementary Fig. S1). Genetic distances ranged from 0.0000% to 0.3477%, with a mean of 0.1047%. The greatest genetic distance was observed between Bursingh Farm (BUR) and Palm/Wallen (PAW), indicating higher sequence divergence between these populations.

Pairwise *F*_ST_ comparisons revealed low to high levels of genetic differentiation among populations (see Supplementary Fig. S1). *F*_ST_ values ranged from –1.0000 to 0.74757, although most comparisons were not statistically significant (*P* > 0.0500). However, significant genetic differentiation (*P* ≤ 0.0500) was detected in 24 pairwise comparisons. The lowest significant *F*_ST_ value was 0.07623, observed between Frome Farm 3 (FF3) and New Yarmouth (NEY), while the highest was 0.36364, between Bursingh Farm (BUR) and Worthy Park (WPK). These results suggest varying degrees of genetic structuring among populations. Several population pairs exhibited low but significant *F*_ST_ values (*P* ≤ 0.0500), indicative of some level of gene flow. In contrast, other pairs showed moderate to high genetic differentiation (*F*_ST_ > 0.25), suggesting restricted gene flow and potential population isolation.

### Genetic and Population Structure

The analysis of molecular variance (AMOVA) indicated that the majority of genetic variation (94.47%) resided within populations, while only 5.53% of the variation was attributed to differences among populations. Overall population differentiation was low but statistically significant (*F*_ST_ = 0.0553, *P* ≤ 0.0500), suggesting weak but detectable population structure. The estimated number of migrants per generation (*N_m_*) was 4.29, exceeding the threshold of 1 and indicating moderate to high levels of gene flow among populations.

The Mantel test revealed a very weak, negative correlation between genetic and geographic distance (*r* = –0.0270), but this relationship was not statistically significant (*P* > 0.0500), suggesting that isolation by distance does not explain the observed genetic patterns.

Neutrality tests further supported demographic expansion. Both Tajima’s *D* (*D* = –2.476) and Fu’s *Fs* (*Fs* = –35.427) were significantly negative (*P* ≤ 0.0500), indicating a departure from neutrality consistent with recent population expansion. Tajima’s *D* values across the 25 sampled populations ranged from –1.609 to 1.757 (Table 1), with 20 localities exhibiting negative values. The most negative and statistically significant value was observed at Palm/Wallen (PAW) (*D* = –1.609, *P* < 0.0500), implying an excess of rare variants indicative of recent expansion. In contrast, Bursingh Farm (BUR) exhibited a positive Tajima’s *D* value (*D *= 1.757), which may reflect a deficit of rare alleles; however, this value was not statistically significant (*P* > 0.0500).

### Regional Genetic Divergence and Affinities of Jamaican *Diatraea saccharalis*

The regional maximum likelihood (ML) phylogenetic tree revealed that Jamaican *D. saccharalis* haplotypes formed a distinct and well-supported clade, clearly separated from most reference sequences originating from other regions of the Americas (Fig. 2). All Jamaican haplotypes were unique to the country, suggesting geographic isolation and local differentiation. However, the Jamaican haplotypes were not entirely divergent, as they exhibited a strong phylogenetic association with sequences from Florida, USA. This grouping was designated the “Caribbean Clade.”

In contrast, sequences from El Salvador, Texas, Louisiana, and Mexico clustered separately into a group designated the “Mesoamerican–Southern US Clade.” South American sequences, specifically those from Brazil, Argentina, and Bolivia, formed a third distinct strongly supported clade (bootstrap value = 70) and referred to as the “South America Clade.”

Within the Jamaican population, Hap_20 and Hap_21 formed a more basal subclade relative to the main Caribbean cluster. Notably, Hap_21 was the most divergent among the Jamaican haplotypes and appeared to share a more recent lineage with haplotypes from Central and South America, suggesting historical connectivity or ancestral polymorphism.

Regional pairwise genetic distance estimates supported the phylogenetic findings (Table 2). The Jamaican population was most closely related to the Florida population, with a genetic distance of 0.6%. Moderate genetic differentiation was observed between Jamaica and populations from Brazil, Bolivia, Argentina (South America), and El Salvador (Central America) (2.3% to 2.6%). The highest genetic distances were recorded between Jamaica and populations from Texas (3.0%), Louisiana (2.9%), and Mexico (2.8%), indicating more substantial genetic divergence from these mainland populations.

**Table 2.** Pairwise percentage genetic distance, based on Kimara’s 2-parameter, among populations of the sugarcane borer, *Diatraea saccharalis*, from various locations, including Texas, Bolivia, Argentina, Florida, El Salvador, Brazil, Mexico, Louisiana, and Jamaica

	Texas	Bolivia	Argentina	Florida	El Salvador	Brazil	Mexico	Louisiana	Jamaica
**Texas**									
**Bolivia**	2.8%								
**Argentina**	2.6%	1.3%							
**Florida**	3.0%	2.6%	2.6%						
**El Salvador**	3.0%	2.4%	2.2%	2.6%					
**Brazil**	3.0%	0.8%	0.5%	2.3%	2.1%				
**Mexico**	3.0%	2.6%	2.4%	2.8%	0.3%	2.2%			
**Louisiana**	3.0%	2.7%	2.4%	2.9%	0.6%	2.3%	0.6%		
**Jamaica**	3.0%	2.4%	2.6%	0.6%	2.6%	2.3%	2.8%	2.9%	

## Discussion

Understanding genetic variation within pest insect populations, such as *D. saccharalis*, is critical for developing effective and sustainable pest management strategies. In the present study, a partial segment of the mitochondrial COI gene from 239 Jamaican sugarcane borer specimens was analyzed to determine genetic diversity parameters and population structure. There was moderate genetic diversity, and 25 haplotypes in Jamaica, with HAP_1 most abundant. Nucleotide composition exhibited a pronounced adenine-thymine (A/T) bias (68.5%), consistent with patterns previously reported for mitochondrial DNA in this species (Li et al. 2011).

The overall genetic diversity observed among Jamaican populations (*h* = 0.392 ± 0.040) was moderate, comparable to values reported from other regional studies (Joyce et al. 2014, Lopes et al. 2014, Francischini et al. 2017, Fogliata et al. 2022). Despite this moderate overall diversity, individual localities displayed considerable variation, ranging from very low to high haplotype diversity. For example, minimal genetic diversity was observed at six sites, including Frome Farm 1 (FF1) and Bengal Farm (BEN), whereas significantly higher diversity was detected at Frome Farm 4 (FF4) and New Yarmouth (NEY).

Sites exhibiting high genetic diversity, such as Frome Farm 4 (FF4) and New Yarmouth (NEY), suggest robust local populations with larger effective population sizes facilitating significant gene flow from surrounding regions. This scenario is particularly evident at NEY, characterized by extensive fertile lands suitable for sugarcane cultivation and surrounded by numerous contiguous sugarcane farms. Additionally, this region consistently experiences high levels of sugarcane borer infestation (Neath et al. 2023a). However, caution must be exercised when interpreting results from FF4, where only 2 individuals were sampled, as limited sample sizes may not accurately represent the genetic diversity present in the broader local population. In particular, genetic parameters such as allelic richness and heterozygosity are known to be especially sensitive to sample size effects (Pruett and Winker 2008, Sinclair and Hobbs 2009).

Conversely, the low genetic diversity observed at specific locations suggests localized phenomena such as genetic bottlenecks, founder effects, or intense selective pressures (Barker et al. 2009). For instance, isolated farms such as Bengal Farm (BEN) (the sole sugarcane-producing site within the parish of St. Ann) (Fig. 1) could experience substantial reductions in effective population size, potentially leading to loss of genetic variation due to limited gene flow and increased genetic drift.

A total of 25 COI haplotypes were identified across all sampled localities, with Hap_1 being the most prevalent, detected in more than 70% of specimens and at all surveyed sites. The considerable number of haplotypes observed underscores a substantial degree of genetic diversity within the Jamaican sugarcane borer population. Nevertheless, the widespread distribution of Hap_1 throughout Jamaica suggests extensive gene flow, indicative of a highly successful lineage with adaptive capabilities enabling it to thrive across diverse environmental conditions. Although adult sugarcane borers exhibit relatively weak flight and limited natural dispersal abilities (Caixeta 2010), the island-wide occurrence of this dominant haplotype may be facilitated by human-mediated activities, such as the transportation of cane seeds and stalks for processing at sugar mills. It has been documented that human-aided dispersal significantly contributes to gene flow in insect pest populations (Peterson and Denno 1998, Bohonak 1999, Francischini et al. 2019).

Furthermore, the identification of 22 haplotypes restricted to single localities (“private haplotypes”) indicates limited gene flow at finer spatial scales and points toward local adaptation to specific environmental conditions (Sjöstrand et al. 2014). The NEY site exemplifies both high haplotype diversity and the presence of unique haplotypes, harboring a total of 7 haplotypes, of which 4 were exclusive to that site. Comparable patterns were reported in Brazilian populations of *D. saccharalis*, reflecting a combination of long-term population stability, substantial gene flow, and expansive, contiguous sugarcane production areas (Francischini et al. 2017).

Phylogenetic analyses revealed no clear geographic structure in the distribution of the Jamaican haplotypes, a pattern corroborated by the low genetic distances among populations and the star-like configuration observed in the haplotype network. These results collectively indicate limited genetic differentiation among populations of the sugarcane borer in Jamaica. A similar lack of geographic differentiation was observed among sugarcane borer populations in El Salvador, although in that case, significant host-plant-associated differentiation was evident (Joyce et al. 2016). Interestingly, our findings align closely with previous reports demonstrating weak genetic structuring of sugarcane borer populations within other countries (Joyce et al. 2014, 2016, Lopes et al. 2014, Fogliata et al. 2022). Such limited genetic differentiation is typically characteristic of populations experiencing high gene flow within a defined geographic area (Gillet and Gregorius 2008).

Despite the overall low differentiation, significant (*P* ≤ 0.0500) pairwise *F*_ST_ values were detected among 24 population comparisons, indicating variability in gene flow across the island. These findings suggest that, although widespread dispersal of the dominant haplotype (Hap_1) occurs, localized pockets of genetic isolation or restricted gene flow persist. Factors contributing to these restrictions likely include the inherently limited flight capabilities of adult sugarcane borers, as previously discussed, coupled with geographical barriers such as mountainous terrain and hills and geographical distance (Silva-Brandão et al. 2015b, Zhang et al. 2015). Notably, the observed pattern of significant genetic differentiation does not strictly correspond to geographical proximity, as both closely situated and geographically distant locations exhibited combinations of low and moderate levels of genetic differentiation. This complexity indicates that gene flow dynamics within Jamaican populations of *D. saccharalis* are likely influenced by multiple interacting ecological and anthropogenic factors.

This interpretation is further supported by the Mantel test, which indicated a very weak, negative correlation (*r* = –0.0270) between genetic and geographic distances that was not statistically significant (*P* > 0.0500). Consequently, isolation by distance (IBD) does not appear to explain the observed genetic patterns within Jamaican populations of *D. saccharalis.* Similarly, Joyce et al. (2016) found no correlation between genetic and geographic distances in sugarcane borer populations from El Salvador. The analysis of molecular variance (AMOVA) indicated that the majority (94.47%) of genetic variation in Jamaican *D. saccharalis* populations occurred within individual populations, with only 5.53% of variation attributed to differences among populations. This result, combined with the low but statistically significant overall *F*_ST_ value (0.05529, *P* ≤ 0.0500), indicates a weak yet detectable level of population structuring across the island. Such population structure is characteristic of pest populations experiencing regular gene flow and frequent exchange of genetic material. Comparable patterns of genetic structuring have been reported in other lepidopteran pests, including the Asian corn borer (*Ostrinia furnacalis*) and European corn borer (*Ostrinia nubilalis*) (Krumm et al. 2008, Li et al. 2010). Similar to our findings, these studies documented low but significant *F*_ST_ values coupled with relatively high estimates of gene flow (*N_m_*). Collectively, these results suggest a scenario in which population differentiation is moderated by consistent gene flow, maintaining genetic cohesion at broader spatial scales. It is important to acknowledge, however, that parameters such as *F*_ST_ and N_m_ while commonly used as indicators of gene flow, rely on assumptions that may not fully reflect natural biological systems (Whitlock and McCauley 1999).

Therefore, to gain further insight into the genetic structure observed in Jamaican populations of *D. saccharalis*, neutrality tests (Tajima’s D and Fu’s Fs) were employed to infer aspects of demographic history, particularly since isolation by distance did not explain the population structuring observed. Both Tajima’s D and Fu’s Fs tests yielded significantly negative values, indicating that the Jamaican sugarcane borer populations have likely undergone a recent demographic expansion. Such expansions typically lead to rapid increases in population size accompanied by the accumulation of rare and novel mutations. This scenario potentially explains the detection of Hap_20 and Hap_21, identified at New Yarmouth (NEY) and Palm/Wallen (PAW), respectively, which were among the most genetically distant haplotypes, forming basal lineages in the maximum likelihood phylogenetic tree. The significantly negative Tajima’s D value observed at PAW further underscores this demographic event.

It is widely hypothesized that *D. saccharalis* originated in the lower Amazon Basin (Myers 1935) and subsequently spread to the Greater Antilles (including Jamaica) where sugarcane became its primary host following reduced availability of its original native host plants (Myers 1932, Francischini et al. 2019). Such historical shifts in host-plant availability could explain the demographic expansion observed in Jamaican populations. As sugarcane cultivation intensified, favorable habitat conditions and abundant host resources likely facilitated rapid increases in the local sugarcane borer populations. Similar demographic expansions have been documented for other lepidopteran pests, including the soybean looper (*Chrysodeixis includens*) and the cotton bollworm *(Helicoverpa armigera*), both of which experienced population expansions coinciding with increases in the cultivation of their respective host plants (Silva et al. 2020, Yang et al. 2025).

Regional analyses conducted using maximum likelihood (ML) phylogenetic reconstruction and pairwise genetic distances revealed clear genetic differentiation of *D. saccharalis* populations across the Americas. Such regional differentiation has been consistently documented in previous studies examining the COI gene in sugarcane borer populations (Joyce et al. 2014, 2016, Lopes et al. 2014, Silva-Brandão et al. 2015a, Francischini et al. 2017, 2019, Fogliata et al. 2022). However, this study represents the first detailed characterization of COI gene variation in *D. saccharalis* populations from the Caribbean region. All Jamaican haplotypes formed a distinct group, termed here as the “Caribbean Clade,” demonstrating a robust phylogenetic association with populations from Florida, USA (Fig. 2). The observed genetic differentiation of Jamaican haplotypes aligns with expectations for island populations, wherein geographic isolation promotes subsequent local genetic divergence (Frankham 1996, Hartl and Clark 1997). Interestingly, the close phylogenetic relationship between the Jamaican and Florida populations suggests a recent or ongoing shared ancestry, potentially facilitated by limited gene flow. Such patterns are frequently observed in island biogeographic systems, where initial colonization from nearby mainland populations is followed by localized divergence (Avise 2000). This has been observed with three noctuid pests in Jamaica. Close genetic relationships were reported with Florida populations (Neath et al. 2023b).

The clear delineation of additional groups, namely the “Central America Clade” and the “Mesoamerican–Southern US Clade,” supports a broader regional genetic structuring of *D. saccharalis* populations across the Americas. These findings contribute to accumulating evidence indicating the presence of cryptic subspecies or distinct geographical strains of *D. saccharalis*. Silva-Brandão et al. (2015a) reported significant genetic structuring correlated with geographic distance in Brazil, indicative of geographically isolated strains within a country. Notably, the genetic types of *D. saccharalis* observed in this study appear to be geographically restricted, with no evidence of haplotype sharing between countries. For instance, neither the Brazilian genotype nor the El Salvadoran haplotype was detected in the Jamaican populations, suggesting limited transboundary movement or recent gene flow of *D. saccharalis* between these regions.

In Jamaica, management of the sugarcane borer is primarily achieved through classical biological control using *Cotesia flavipes*, and field resistant commercial sugarcane varieties (Neath et al. 2025). The findings from this study present important considerations for improving pest management strategies in a production a system where chemical control is seldom used (Bellini and Dolinski 2012). The evidence of substantial gene flow across populations suggests that any emergence of pesticide resistance could potentially spread rapidly throughout sugarcane-growing regions of the island. This underscores the need for proactive resistance management and careful consideration before the introduction of chemical control options. Conversely, the presence of unique haplotypes at specific sites highlights the potential for site-specific management approaches. Localized genetic differentiation may reflect environmental pressures or adaptation, offering an opportunity to develop tailored control strategies that account for the genetic composition of regional populations and associated agroecological conditions.

This study presents the first comprehensive assessment of the genetic diversity, population structure, and regional phylogeographic relationships of *D. saccharalis* in Jamaica using mitochondrial COI markers. The results reveal moderate genetic diversity, limited population differentiation, and strong signals of recent demographic expansion, likely driven by the intensification of sugarcane cultivation and human-mediated dispersal. The identification of a distinct Caribbean clade, closely aligned with populations in Florida, adds to growing evidence of regional genetic structuring of *D. saccharalis* across the Americas. While mitochondrial COI data have provided a useful foundation, incorporating nuclear markers (such as microsatellites or SNPs, along with COII) could offer complementary insights and enhance the resolution of population genetic structure (Francischini et al. 2019, Fogliata et al. 2022). Additionally, expanding genetic analyses to include *D. saccharalis* populations from other Caribbean countries would also contribute to a more comprehensive understanding of regional diversity and facilitate the tracking of historical and contemporary dispersal patterns throughout the Caribbean basin.

## Supplementary Material

toag111_Supplementary_Data
