# Supplementary material for: Mitochondrial haplotype diversity and population dynamics of the sugarcane borer, Diatraea saccharalis (Lepidoptera: Crambidae), in Jamaica
Source: J Econ Entomol. 2026 Apr 27;119(3):2284–94. doi: 10.1093/jee/toag111 (PMC13268523; doi:10.1093/jee/toag111)
Supplement: toag111_Supplementary_Data [file toag111_supplementary_data.zip › Supplemental Figure 1.docx]

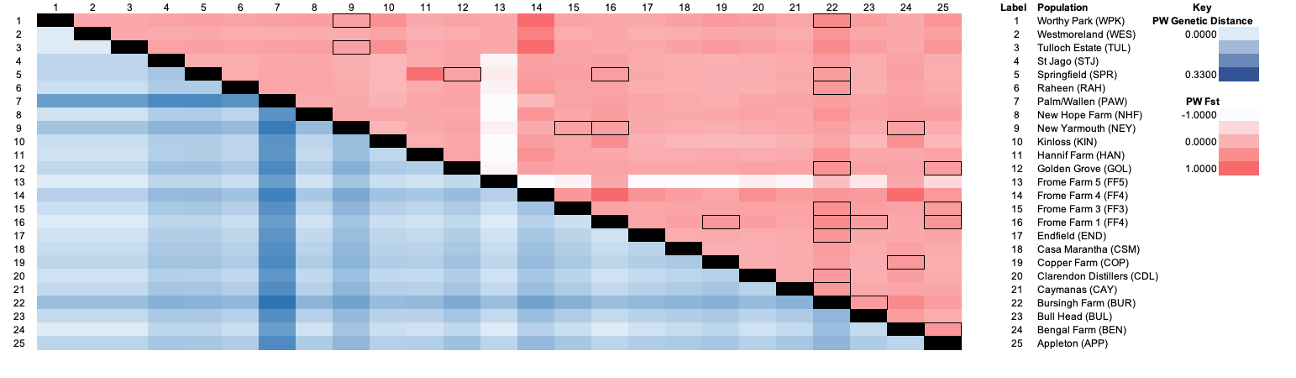


Heatmap illustrating pairwise genetic distances (left), based on Kimura’s 2-paramter and pairwise *F_ST_* values (right) among populations of the sugarcane borer, Diatraea saccharalis, sampled from 25 locations in Jamaica. Genetic distances are represented by shades of blue, with darker shades indicating greater genetic distance, while pairwise *F_ST_* values are represented by shades of red, with darker shades indicating higher genetic differentiation. Black cells along the diagonal represent comparisons of identical populations. Cells with black borders indicate significant (P ≤ 0.0500) *F_ST_* comparisons. PW = pairwise
